# A Synopsis of the Practice of Medicine

**Published:** 1894-12

**Authors:** 


A Synopsis of the Practice of Medicine. For Practitioners and Students. By William Blair Stewart, A. M., M. D., Lecturer on Therapeutics; late instructor on Practice of Medicine in the Medico-Chirurgical College of Philadelphia; Demonstrator in the Philadelphia School of Anatomy, etc. New York: E. B. Treat, Publisher, 5 Cooper Union. One large octavo volume, about 434 pages. Cloth, $2.75.

This work has been undertaken after several years of experience by the author as instructor on the subject of the Practice of Medicine, his purpose being to prepare and present to the profession a brief synopsis of the subject, not with the view of replacing the expensive and elaborate publications, but to give to the busy practitioner and student, at a small cost, concise and accurate descriptions which will suggest outlines and practical thoughts upon etiology, symptomology, pathology, diagnosis, prognosis, and treatment.

The author has used every endeavor to obtain the best material from every reliable source. All of the prominent authorities in the recently issued text-books and systems, also the current medical literature, have been laid under contribution, and the most approved methods of treatment have been given prominence. Many drugs and methods have not been considered at length, not on account of their inutility, but from the fact that better forms of treatment have taken their place.

